# Supplementary material for: Functional Assessments of Gynecologic Cancer Models Highlight Differences Between Single-Node Inhibitors of the PI3K/AKT/mTOR Pathway and a Pan-PI3K/mTOR Inhibitor, Gedatolisib
Source: Cancers (Basel). 2024 Oct 17;16(20):3520. doi: 10.3390/cancers16203520 (PMC11505998; doi:10.3390/cancers16203520)
Supplement: Supplementary file 1 [file cancers-16-03520-s001.zip › Supplementary Figures.pdf]

Supplementary Figures for

Functional Assessments of Gynecologic Cancer Models Highlight Differences Between Single-Node Inhibitors of the PI3K/AKT/mTOR Pathway and a Pan-PI3K/mTOR Inhibitor, Gedatolisib

Aaron Broege, Stefano Rossetti *, Adrish Sen, Arul S. Menon, Ian MacNeil, Jhomary Molden and Lance Laing *

* Correspondence: srossetti@celcuity.com (S.R.); llaing@celcuity.com (L.L.)

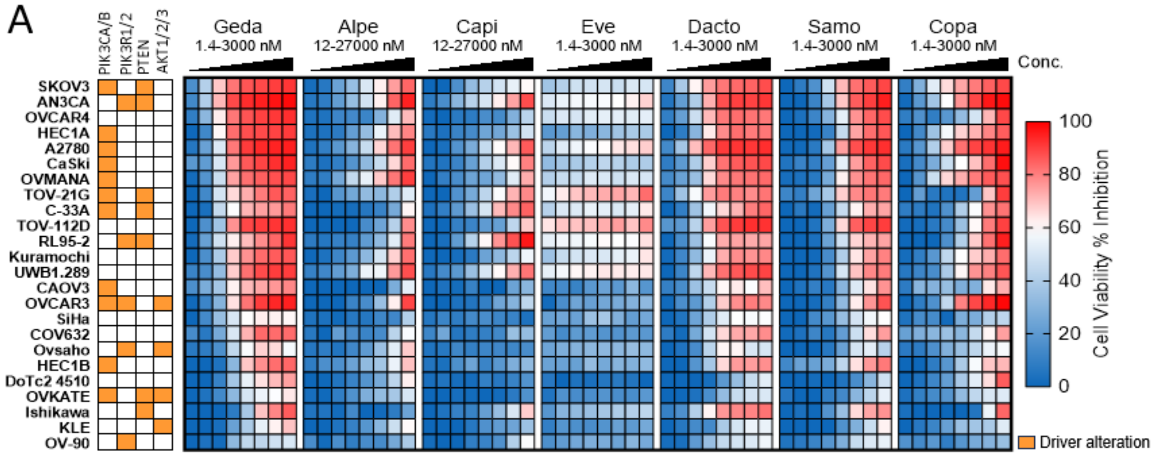

B

	PIK3CA/B	PIK3R1/2	PTEN	AKT1/2/3	Viability IC50 (nM)							Viability % inhibition at 333 nM						
					Geda	Alpe	Capi	Eve	Dacto	Samo	Copa	Geda	Alpe	Capi	Eve	Dacto	Samo	Copa
SKOV3	X		X		5.7	993	6000	Unst	7	43	13	88%	34%	33%	47%	83%	82%	81%
AN3CA		X	X		5.8	1154	917	1.9	15	58	24	95%	34%	42%	60%	90%	85%	89%
OVCAR4					7.9	10299	27000*	4.7	13	91	353	90%	13%	8%	53%	83%	77%	49%
HEC1A	X				10	2338	27000*	3000*	22	133	26	88%	21%	14%	36%	83%	74%	72%
A2780	X				11	1169	1458	4	6	52	18	94%	30%	27%	64%	92%	88%	89%
CaSki	X				12	3578	2749	3000*	17	104	62	89%	17%	22%	42%	85%	75%	70%
OVMANA	X				13	667	7756	Unst	19	75	15	85%	45%	24%	48%	81%	77%	80%
TOV-21G	X		X		13	18000	1483	0.6	8	51	652	81%	25%	26%	74%	80%	78%	21%
C-33A	X		X		16	20799	868	0.5	27	162	197	75%	2%	35%	51%	72%	62%	59%
TOV-112D					16	11138	13079	0.2	13	70	213	91%	0%	11%	75%	90%	85%	61%
RL95-2		X	X		17	8345	572	0.7	22	124	164	78%	10%	42%	60%	74%	67%	68%
Kuramochi					18	3152	14502	22	27	170	61	83%	19%	7%	53%	78%	64%	68%
UWB1.289					20	1379	1245	1.2	16	120	64	88%	28%	34%	63%	86%	69%	77%
CAOV3	X				21	11837	27000*	3000*	45	367	494	74%	-4%	10%	19%	63%	51%	28%
OVCAR3	X	X		X	24	5589	27000*	3000*	49	226	42	90%	11%	5%	26%	77%	63%	89%
SiHa					29	27000*	27000*	3000*	65	351	3000*	63%	6%	13%	28%	60%	49%	27%
COV632					36	17675	27000*	3000*	38	424	384	83%	15%	8%	17%	73%	47%	45%
Ovsaho		X		X	52	9377	27000*	3000*	52	Unst.	588	67%	12%	9%	31%	62%	49%	43%
HEC1B	X				60	6174	27000*	3000*	53	141	179	78%	23%	24%	30%	76%	65%	58%
DoTc2 4510					72	12988	27000*	3000*	1831	2860	264	65%	11%	2%	7%	34%	14%	53%
OVKATE	X		X	X	120	27000	27000*	3000*	1875	1678	1234	55%	16%	1%	15%	46%	31%	44%
Ishikawa			X		157	27000*	8063	3000*	19	185	918	69%	13%	10%	39%	79%	66%	5%
KLE				X	212	11161	27000*	3000*	470	1115	Unst	54%	10%	6%	23%	49%	28%	37%
OV-90		X			Unst	27000*	16900	3000*	240	778	3000*	44%	8%	9%	24%	49%	46%	26%
**Averages**																		
All Cell Lines (n=24)					41	11076	15525	1774	206	408	520	77.8%	16.6%	17.6%	41.1%	72.7%	62.1%	55.8%
PAM not altered (n=7)					28	11947	19547	1290	286	584	620	80.3%	13.1%	11.9%	42.5%	72.0%	57.9%	54.2%
PAM altered (n=17)					47	10717	13869	2001	173	331	477	76.7%	18.1%	19.9%	40.5%	72.9%	63.9%	56.4%
PIK3CA/B altered (n=11)					28	8922	14119	2001	193	276	267	81.5%	20.1%	20.1%	41.0%	76.1%	67.8%	62.8%
PIK3R1/2 altered (n=6)					51	13078	13409	2000	66	274	789	73.9%	14.7%	19.6%	40.1%	71.8%	62.5%	53.3%
PTEN altered (n=7)					48	14756	6415	1001	282	329	457	77.4%	19.1%	27.1%	49.3%	74.7%	67.2%	52.3%
AKT1/2/3 altered (n=4)					102	13282	27000	3000	612	1006	621	66.6%	12.3%	5.5%	23.7%	58.6%	42.7%	53.2%
**Medians**																		
All Cell Lines (n=24)					18	9838	15701	3000	24	141	197	81.9%	13.9%	12.2%	40.7%	77.6%	65.1%	58.5%
PAM not altered (n=7)					20	11138	27000	22	27	170	264	83.3%	13.1%	8.1%	53.2%	77.7%	63.8%	52.6%
PAM altered (n=17)					16	8345	8063	3000	22	137	172	78.0%	16.4%	22.3%	39.4%	77.4%	65.6%	59.0%
PIK3CA/B altered (n=11)					13	5589	7756	3000	22	133	62	85.0%	21.2%	23.6%	42.0%	79.6%	74.2%	69.6%
PIK3R1/2 altered (n=6)					24	8861	12482	3000	35	185	376	73.7%	11.6%	9.9%	35.4%	75.8%	64.2%	55.6%
PTEN altered (n=7)					16	18000	1483	1	19	124	197	78.0%	16.4%	33.2%	50.9%	78.7%	66.6%	59.0%
AKT1/2/3 altered (n=4)					86	10269	27000	3000	261	1115	588	61.2%	11.6%	5.6%	24.4%	55.4%	39.9%	43.7%

Supplementary Figure S1. Assessment of PAM inhibitors response in gynecologic cancer cell lines using endpoint cell viability analyses. **A**. Cell viability was assessed at the end of a 72-hours treatment with gedatolisib and other PAM inhibitors by RTGlo MT assay, and the % inhibition was calculated relative to DMSO-treated cells (se t as 1). The cell viability % inhibition in 24 gynecologic cancer cell lines is shown in a heatmap. See Supplementary Table 6 for data. **B.** IC50 values and % inhibition of cell viability in response to 333 nM PAM inhibitors in the panel of 24 gynecologic cancer cell lines. Average and median values in subpopulations with or without altered PAM pathway genes are shown. * = Max concentration tested, IC50 not reached; Unst. = unstable due to poor curve fitting that prevented reliable IC50 calculation.

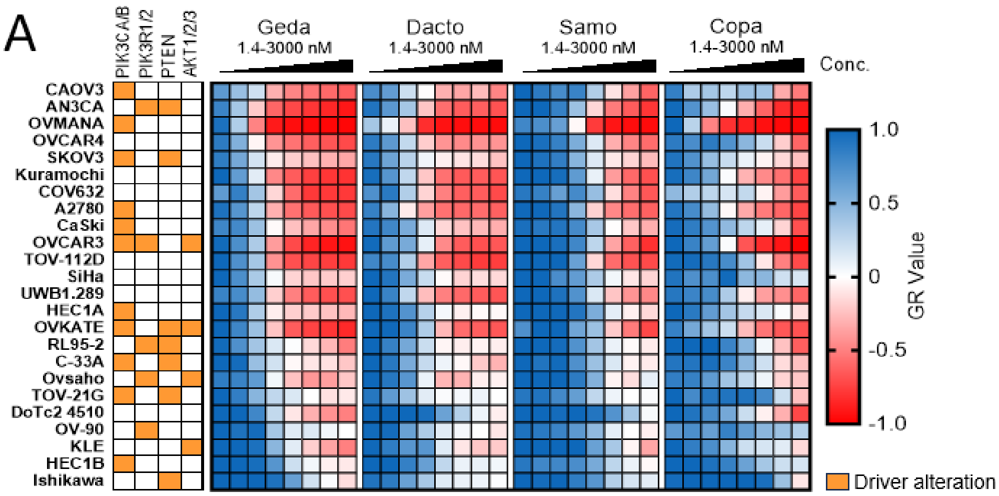

B

	PIK3CA/B	PIK3R1/2	PTEN	AKT1/2/3	GR50 (nM) Geda	GR50 (nM) Dacto	GR50 (nM) Samo	GR50 (nM) Copa	GR-Max Geda	GR-Max Dacto	GR-Max Samo	GR-Max Copa	GR-AOC Geda	GR-AOC Dacto	GR-AOC Samo	GR-AOC Copa
CAOV3	X				2.3	5	70	15	-0.6	-0.5	-0.6	-0.5	4.1	3.5	2.2	2.7
AN3CA		X	X		3.3	8	32	11	-0.9	-0.7	-0.8	-0.9	4.7	3.8	2.8	3.6
OVMANA	X				3.6	1	13	4	-1.0	-1.0	-1.0	-1.0	5.2	5.5	4.1	5.0
OVCAR4					4.2	7	44	100	-0.7	-0.4	-0.6	-0.6	4.2	3.6	2.4	2.0
SKOV3	X		X		5.1	7	39	12	-0.3	-0.2	-0.3	-0.3	3.3	2.9	2.1	2.6
Kuramochi					6.4	8	55	13	-0.8	-0.7	-0.7	-0.7	4.2	3.8	2.4	3.2
COV632					7.1	9	59	3	-0.8	-0.7	-0.7	-0.6	4.1	3.8	2.4	3.3
A2780	X				7.2	4	36	10	-0.7	-0.6	-0.6	-0.7	3.9	4.2	2.7	3.5
CaSki	X				8.5	13	80	49	-0.5	-0.3	-0.3	-0.7	3.3	2.8	1.9	2.2
OVCAR3	X	X		X	8.7	12	75	16	-0.9	-0.7	-0.8	-1.0	4.2	3.5	2.2	3.7
TOV-112D					8.9	7	48	84	-0.8	-0.8	-0.7	-0.7	4.0	4.1	2.4	2.2
SiHa					9.0	16	65	192	-0.2	-0.1	-0.2	0.2	2.8	2.4	1.7	1.3
UWB1.289					9.0	8	60	24	-0.7	-0.7	-0.5	-0.6	3.8	3.7	2.1	2.8
HEC1A	X				9.5	22	131	29	-0.3	0.0	-0.2	-0.1	2.9	2.2	1.4	2.0
OVKATE	X		X	X	10.9	28	74	6	-0.8	-0.7	-0.7	-0.7	3.8	2.9	2.3	3.6
RL95-2		X	X		12.2	15	87	121	-0.5	-0.1	-0.1	-0.6	2.8	2.4	1.5	1.7
C-33A	X		X		13.2	18	100	117	-0.2	-0.3	-0.1	-0.3	2.5	2.5	1.6	1.7
Ovsaho		X		X	15.0	17	109	29	-0.4	-0.1	-0.1	-0.2	3.0	2.6	1.4	2.3
TOV-21G	X		X		15.6	8	49	603	-0.1	0.0	0.0	-0.2	2.3	2.5	1.9	1.0
DoTc2 4510					26.1	182	823	56	-0.5	-0.1	0.0	-0.7	2.4	1.0	0.6	2.1
OV-90		X			31.1	22	78	388	0.0	-0.1	-0.1	0.3	1.7	2.1	1.7	1.4
KLE				X	44.4	38	242	38	-0.5	-0.2	-0.4	-0.2	2.2	2.2	1.1	1.9
HEC1B	X				59.5	51	109	174	-0.1	0.0	0.0	0.2	1.6	1.6	1.7	1.1
Ishikawa			X		158.5	20	200	900	-0.1	0.0	0.1	-0.1	1.2	2.3	1.2	0.4
**Averages**																
All Cell Lines (n=24)					20	22	112	125	-0.5	-0.4	-0.4	-0.5	3.3	3.0	2.0	2.4
PAM not altered (n=7)					10	34	165	67	-0.6	-0.5	-0.5	-0.6	3.6	3.2	2.0	2.4
PAM altered (n=17)					24	17	90	148	-0.5	-0.3	-0.3	-0.4	3.1	2.9	2.0	2.4
PIK3CA/B altered (n=11)					13	15	71	94	-0.5	-0.4	-0.4	-0.5	3.4	3.1	2.2	2.7
PIK3R1/2 altered (n=6)					38	16	97	244	-0.5	-0.3	-0.3	-0.4	2.9	2.8	1.8	2.2
PTEN altered (n=7)					31	15	83	253	-0.4	-0.3	-0.3	-0.4	2.9	2.8	1.9	2.1
AKT1/2/3 altered (n=4)					20	24	125	22	-0.7	-0.4	-0.5	-0.5	3.3	2.8	1.8	2.9
**Medians**																
All Cell Lines (n=24)					9	13	72	34	-0.5	-0.3	-0.3	-0.6	3.3	2.9	2.0	2.2
PAM not altered (n=7)					9	8	59	56	-0.7	-0.7	-0.6	-0.6	4.0	3.7	2.4	2.2
PAM altered (n=17)					11	15	78	29	-0.5	-0.2	-0.3	-0.3	3.0	2.6	1.9	2.2
PIK3CA/B altered (n=11)					9	12	74	16	-0.5	-0.3	-0.3	-0.5	3.3	2.9	2.1	2.6
PIK3R1/2 altered (n=6)					14	16	82	75	-0.4	-0.1	-0.1	-0.4	2.9	2.5	1.6	2.0
PTEN altered (n=7)					12	15	74	117	-0.3	-0.2	-0.1	-0.3	2.8	2.5	1.9	1.7
AKT1/2/3 altered (n=4)					13	22	92	23	-0.7	-0.4	-0.5	-0.5	3.4	2.8	1.8	2.9

Supplementary Figure S2. Analysis of PAM inhibitors response in gynecologic cancer cell lines using growth rate metrics. **A.** Heatmap showing GR values in 24 gynecologic cancer cell lines treated with increasing concentrations of gedatolisib, dactolisib, copanlisib, or samotolisib for 72 hours. See Supplementary Table 7 for data. **B.** GR50, GRMax and GRAOC values for gedatolisib and the other PAM inhibitors in gynecologic cancer cell lines. Average and median values in subpopulations with or without altered PAM pathway genes are shown.

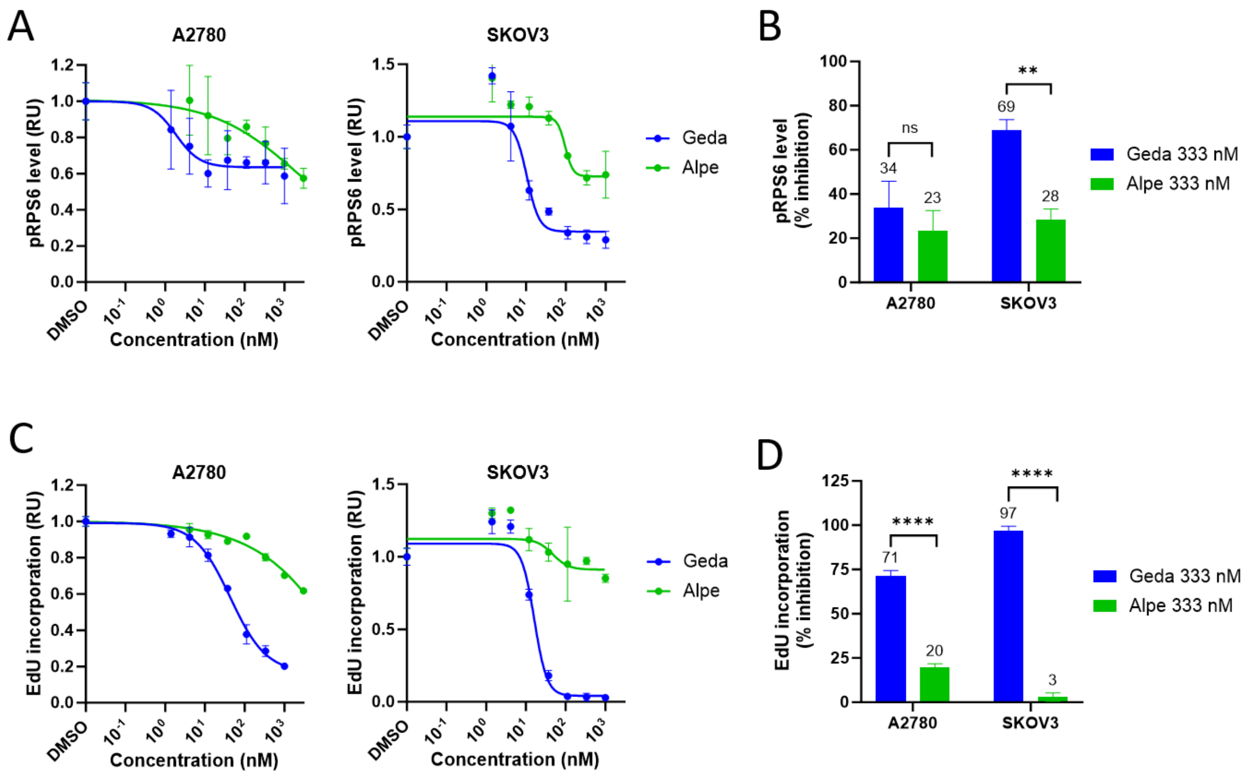

Supplementary Figure S3. Analysis of PAM pathway activity and DNA replication in response to PAM inhibitors in OC cell lines. **A.** PAM pathway activity in A2780 and SKOV3 OC cell lines treated with gedatolisib or alpelisib for 48 hours was assessed by flow cytometry analysis of pRPS6(S235/S236) levels. The median fluorescence intensities (MFI) was normalized to DMSO-treated cells (set as 1) and used to plot dose response curves (DRCs). Data represent mean +/- SD (n=2-6 biologically independent samples). B. Inhibition of pRPS6 in response to 333 nM gedatolisib or alpelisib. Data represent mean +/- SD (n=2 biologically independent samples). ns = not significant; ** $p < 0.01$ by one-way ANOVA Fisher's LSD test. **C.** DNA replication in A2780 and SKOV3 OC cell lines treated with gedatolisib or alpelisib for 48 hours was assessed by flow cytometry analysis of EdU incorporation. The % of EdU+ cells was normalized to DMSO-treated cells (set as 1) and used to plot dose response curves (DRCs). Data represent mean +/- SD (n=2 biologically independent samples). D. Inhibition of EDU incorporation in response to 333 nM gedatolisib or alpelisib. Data represent mean +/- SD (n=2-6 biologically independent samples). **** $p < 0.0001$ by one-way ANOVA Fisher's LSD test.

A

Sample	Age	Ethnicity	Cancer	Subtype	Stage
C1944	52	Caucasian	Ovarian Cancer	High grade serous	pT3c N1
C1966	58	Caucasian	Ovarian Cancer	High grade serous	pT3c N1
C1735	62	Caucasian	Ovarian Cancer	High grade serous	pT3a Nx
C2131	60	Caucasian	Ovarian Cancer	Clear cells	pT2a N0

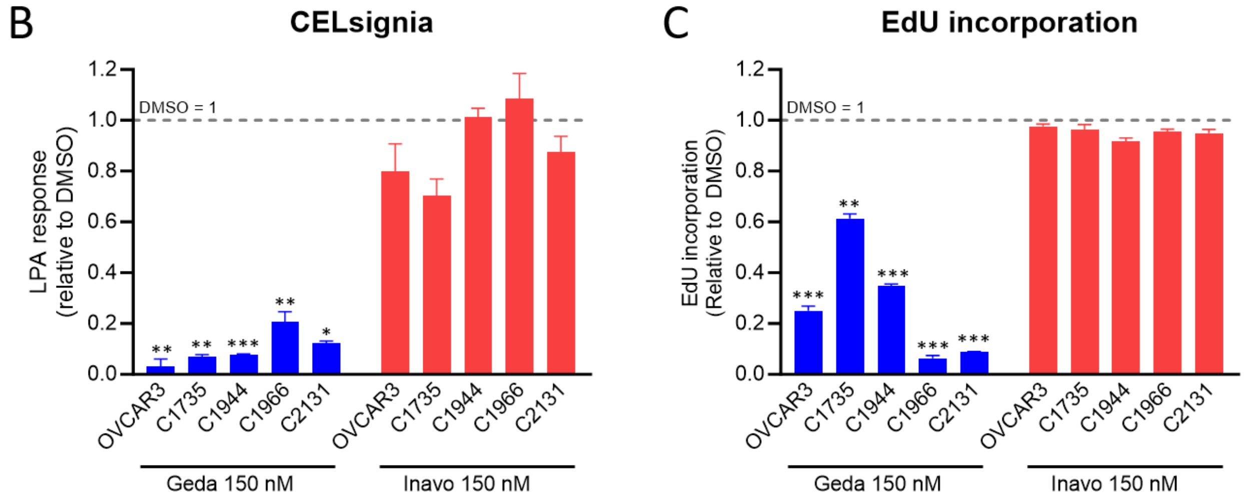

Supplementary Figure S4. Analysis of PAM pathway activity and DNA replication by CELsignia and EdU incorporation in OC cells. **A.** Characteristics of the primary OC cultures tested. **B.** LPA is a well-known oncogenic GPCR agonist that can activate the PAM pathway (reviewed in Balijepalli et al., 2021, Cells, 10(8):2059. doi: 10.3390/cells10082059). The CELSignia test shows that LPA-induced impedance (indicative of stimulation of PAM pathway activity) is inhibited more effectively by gedatolisib than inavolisib in four OC primary cultures and in the OVCAR3 cell line. Data represent mean +/- standard deviation (n=2 biologically independent samples). **C.** EdU incorporation assay showing that treatment with gedatolisib (150 nM, 48 hours) inhibits DNA replication more effectively than invaolisib (150 nM, 48 hours) in four OC primary cultures and in the OVCAR3 OC cell line. Data represent mean +/- standard deviation (n=2 biologically independent samples). * p<0.05, ** p<0.01, *** p<0.001, two-sided unpaired t-test relative to DMSO-treated cells.

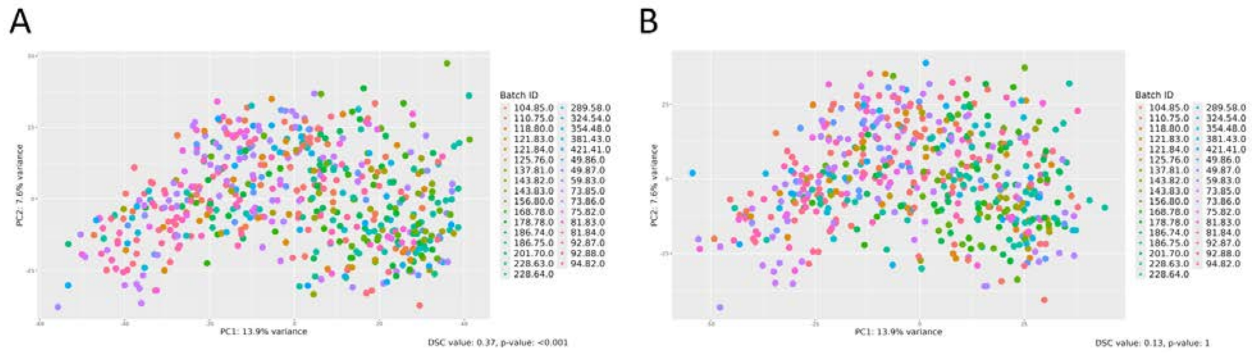

Supplementary Figure S5. PCA plots showing clustering of the filtered and transformed EC RNA-seq data before (A) and after (B) performing batch correction.

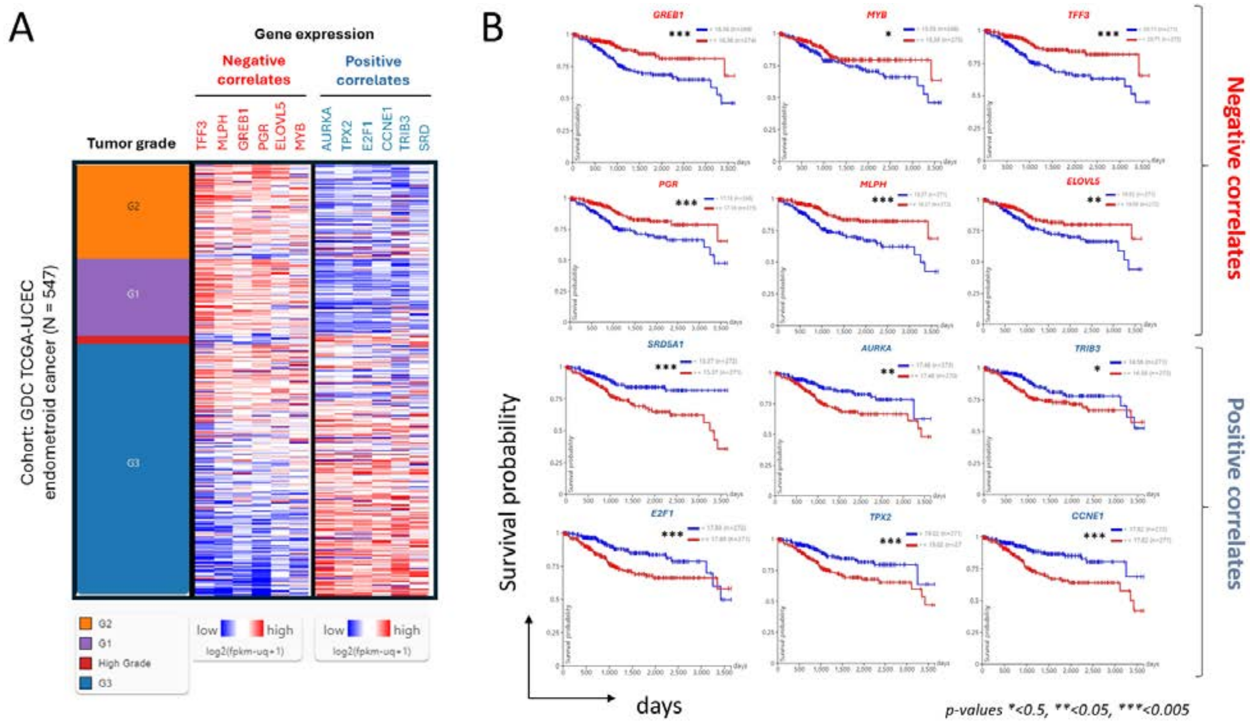

Supplementary Figure S6. Validation of selected mRNAs mapped along the pseudotime-based disease trajectory using Kaplan-Meier analysis of survival possibility. A. Heatmap of representative transcripts that significantly correlate with inferred pseudotime (marked with asterisks in panel D of Figure 7) showing FPKM-UQ normalized gene expression values from 547 TCGA EnC patients (B). Higher expression of transcripts positively correlated with pseudotime is associated with a significantly worse 10-year overall survival in TCGA endometroid cancer patients, whereas higher expression of negatively pseudotime-correlated transcripts predicts significantly better outcomes.

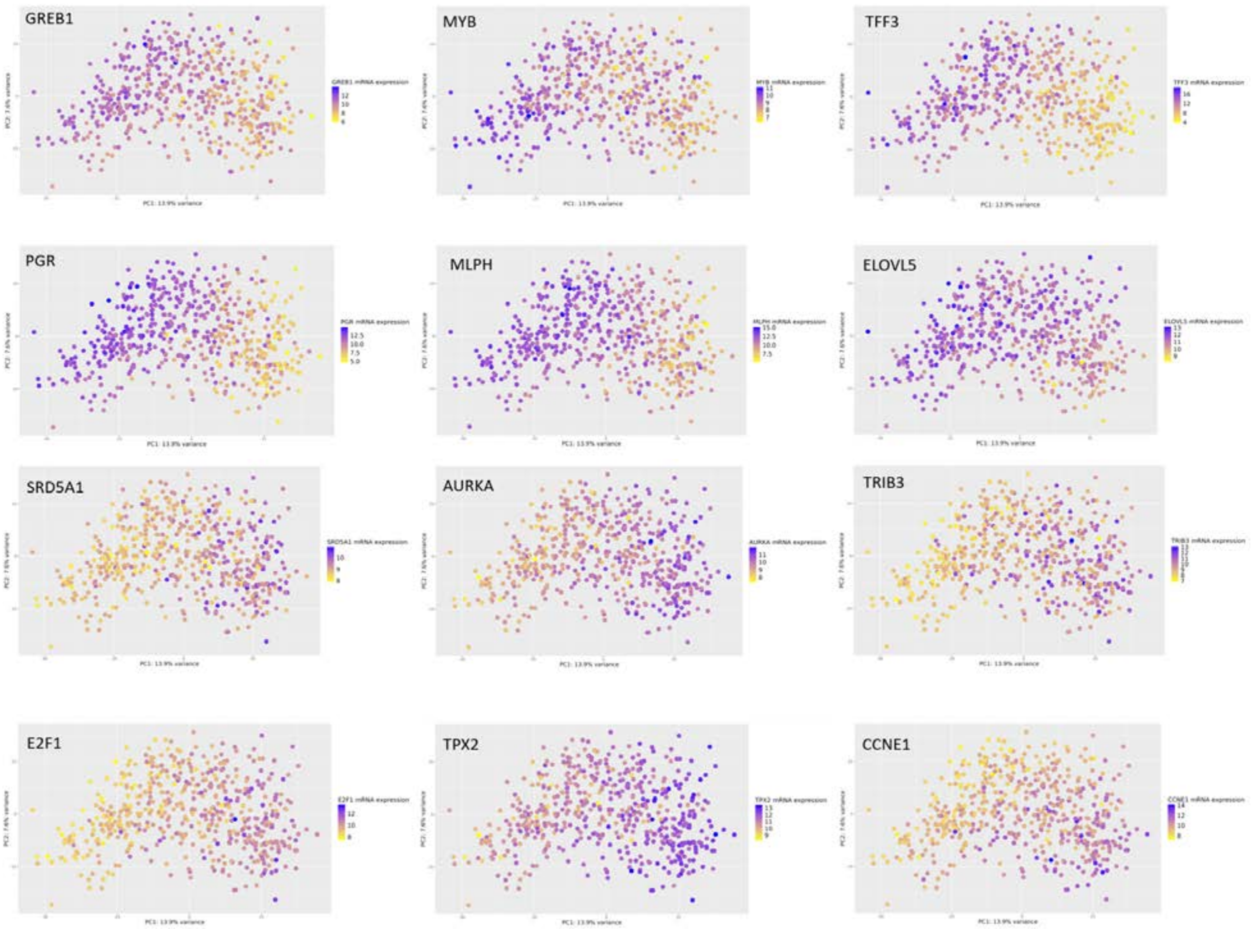

Supplementary Figure S7. PCA plots showing the expression levels of mRNAs selected for Kaplan-Meier analysis (in Supplementary Figure S6) for tumor samples on the pseudotime-based EC trajectory.
